# The Influence of Alternative Fillers on the Adhesive Properties of Mastics Fabricated with Red Mud

**DOI:** 10.3390/ma13020484

**Published:** 2020-01-19

**Authors:** Mayara S. Siverio Lima, Liseane P. Thives, Viktors Haritonovs, Florian Gschösser

**Affiliations:** 1 Faculty of Engineering Science, University of Innsbruck, Technikerstraße 13, 6020 Innsbruck, Austria; florian.gschoesser@uibk.ac.at; 2 Department of Civil Engineering, Federal University of Santa Catarina, Florianópolis-SC 88040-900, Brazil; liseane.thives@ufsc.br; 3 Department of Constructions, Riga Technical University, LV-1658 Riga, Latvia; viktors.haritonovs@rtu.lv

**Keywords:** red mud, fly ash, mastics, adhesive properties

## Abstract

The adhesion between bitumen and aggregates strongly influences the lifetime of pavements. To improve adhesiveness, the road construction industry has been using additives to alter the interfacial energy and improve the affinity of materials in the presence of water. However, the water sensitivity varies according to the mixture design, since the interaction may occur differently depending on the materials chosen. As the use of alternative materials is increasing in road constructions, further analysis of its affinity with aggregates and bitumen is necessary. In that sense, this study evaluates the adhesion performance of mastics mixed with traditional fillers, such as limestone and dolomite, and residues, such as fly ash and red mud. To assess possible interactions with the red mud, the fillers are mixed in distinct percentages and tested for adhesiveness, wettability, penetration, and softening point. The results show the importance of hydrophilicity, asphalt viscosity, and physical–chemical properties to define adhesive interactions.

## 1. Introduction

In order to reduce costs and environmental impacts, a variety of alternative raw materials have been suggested to compose asphalt mixtures (e.g., Portland cement, brick powder, waste glass, steel slag, red mud [1,2] and fly ash).

Red mud, for example, is a solid waste generated during aluminum production [3] and several studies have investigated its use in construction materials [4], such as ceramics [5,6,7,8], bricks [9,10], tiles [11], cement [12,13,14], and in road pavement layers [15,16]. However, its high alkalinity is still a limitation for large-scale applications.

Designing asphalt materials with residues can be challenging, since chemical and physical properties must be previously evaluated in order to ensure the best bitumen–filler–aggregates interaction. 

Taking into account that the mastics (bitumen + filler) are responsible for covering and connecting the aggregates, ensuring the compaction, impermeability, and workability of asphalt mixtures, the affinity between materials must be carefully assessed.

The affinity between bitumen and aggregates has been studied for a long period. Many authors have reported asphalt viscosity, polarity, bitumen composition, chemical, and physical properties such as hydrophilicity, alkalinity, granulometry, shape, and texture as some of the characteristics that significantly affect the adhesion between mineral aggregates and asphalt. Among all, there is no agreement on which properties have more influence.

A number of studies [17,18,19,20] have mentioned the importance of additional properties to be evaluated, such as gradation, shape, and the texture of particles, since they can affect the bitumen–filler–aggregates interaction, whether in dry or wet conditions. Some authors have defended the positions that properties can be assessed by the Rigden voids and bitumen number test methods [19,20]. 

Vassaux et al. [21] identified the most impacting factors that influence the bitumen–aggregate interface. They classified fillers into two main groups: smooth fillers–silica- or calcium-based fillers– and heterogeneous fillers—fillers with a more complex chemical composition. According to the authors, ‘smooth fillers’ are more influenced by the asphalt viscosity, polarity, and saturate content, while ‘heterogeneous fillers’ are influenced by the asphalt viscosity and asphaltenes content.

The selection of filler influences the mastic properties and this study evaluates the adhesive capacity of residues (i.e., red mud and fly ash) and traditional materials (limestone and dolomite) testing different proportions and combinations. In this study, hydrophobic and viscosity tests are performed to evaluate the influence of these properties within adhesivity.

## 2. Materials and Methods 

The red mud used in this research was supplied by a Brazilian alumina refinery (Norsk Hydro Company, Belém-PA, Brazil). Granite aggregate and other fillers were obtained in Latvia. The majority of the tests were performed at the Latvian State Road Laboratory located in Riga.

To analyze the influence of fillers in bituminous mastics, four types were used: limestone (LS), dolomite (DL), fly ash (FA) and red mud (RM). Their characterization was made using different methods, described as follows.

### 2.1. Fillers

To characterize and produce the mastics, firstly, the fillers were dried at 110 °C. Afterwards, they were tested for density, specific surface area (BET method), particle size, Rigden voids, Ph, and chemical composition (XRF). The results are presented in Table 1.

**Figure 1:**
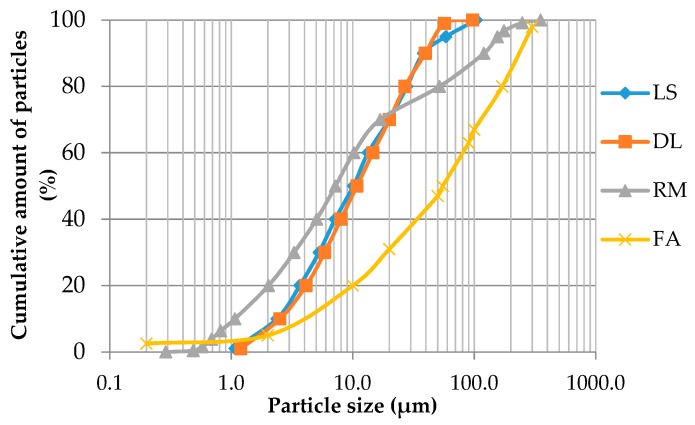
Particle size distribution of different fillers.

The laser particle sizing analysis was performed using the Analysette 22 NanoTec, with a detection range between 0.1 and 1000 μm. Figure 1 shows the granulometry results of the fillers.

The filler chemical analysis was made with an X-ray fluorescence test, using as reference the international standard ISO 12677 [25]. The results are shown in Table 2.

### 2.2. Bitumen

The bitumen 50/70, used to produce asphalt mixtures, was provided by the LOTOS ASFALT Company (Gdańsk, Poland).

To characterize the unaged bitumen, penetration, softening point, Frass breaking point, ductility, and viscosity tests were performed. The bitumen was aged using the rolling thin film oven test (RTFOT) and evaluated for penetration and softening point tests. The results are presented in Table 3.

### 2.3. Mastics 

To evaluate the interactions between fillers, dolomite (DL), limestone (LS), and fly ash (FA) were combined with red mud (RM) and tested in different percentages. The amount of filler took into account the total volume of bitumen. The percentages used were:
• 40% RM • 40% LS • 40% DL • 40% FA • 20% RM + 20% LS • 20% RM + 20% DL • 20% RM + 20% FA   • 20% RM • 20% LS • 20% DL • 20% FA • 10% RM + 10% LS • 10% RM + 10% DL • 10% RM + 10% FA

As red mud has some agglomeration issues, the residue was sifted in a 0.075 mm sieve after drying. This specific sieve was used to guarantee the filler granulometry according to Brazilian standards [33], which consider materials with 65% or more passing in a 0.075 mm sieve. In addition to drying, no additional pre-treatments were required for the other fillers.

To produce the mastics, the bitumen 50/70 was heated at 155 °C and the fillers at 160 °C. These temperatures were designed according to EN 12697-35 [34]. This standard establishes that the compaction temperature should be 150 °C for 50/70 bitumen. The mixing temperature should not be more than 20 °C above the compaction temperature, and aggregates/fillers needed to be heated at a mixing temperature of ±5 °C.

The bitumen and fillers were heated to the pre-set temperatures and mixed by hand using a glass stick for approximately 3–4 min, ensuring the full homogeneity of the mastic.

The mastic properties were evaluated by penetration and softening point tests (before and after RTFOT). The penetration and softening point tests were made to foresee the viscosity of mastics. These tests were chosen instead of the proper viscosity test since the equipment available was not suitable to assess materials with higher viscosities. In the end, it was intended to correlate ‘viscosity’ with the adhesive properties of mastics.

The wettability test was performed by measuring the contact angle between the surface of the mastics and water. A contact angle greater than 90° implied a low affinity and the material was considered hydrophobic. If the liquid spread onto the surface, the contact angle was under 90°, which implied a greater affinity between materials.

### 2.4. Adhesion Test

The test followed the EN 12697-11 standard and used the boiling water stripping method in an adapted form (i.e., without chemicals).

To evaluate the adhesiveness, granite aggregates were used in a size fraction of 8–11 mm, heated at 110 °C for 3 h. After drying, the aggregates were heated at 160 °C for 3 more hours. Bitumen (50/70) and mastics were heated at 155 °C, also for 3 h. The bowl used to mix all the materials was heated with aggregates to avoid losing temperature easily. For each mixture, 510 g of aggregates were used. 

The typical amount of bitumen used to recover the aggregates is 15.6 g. This value is calculated using an equation [35] that considers the relation between the density and size of the aggregates. 

For mastics, this amount must be raised, since the amount and type of filler made the bitumen stiffer. Thus, the amount inserted ranged from 16 to 20 g, approximately. Aggregates and mastics were mixed by hand until the aggregates were fully covered.

Once the mixtures were finished, all the covered aggregates were spread in an aluminum paper to cool down for 12 h at 20 ± 5 °C. Thirty particles of aggregate were carefully chosen, ensuring that their full area was covered.

The particles were allocated in supporting netting with hooks. A beaker with 700 mL of water was put on an electric stove regulated to maintain the water at 100 °C. When the water was boiling, the supporting netting with aggregates was inserted for 30 min.

**Figure 2:**
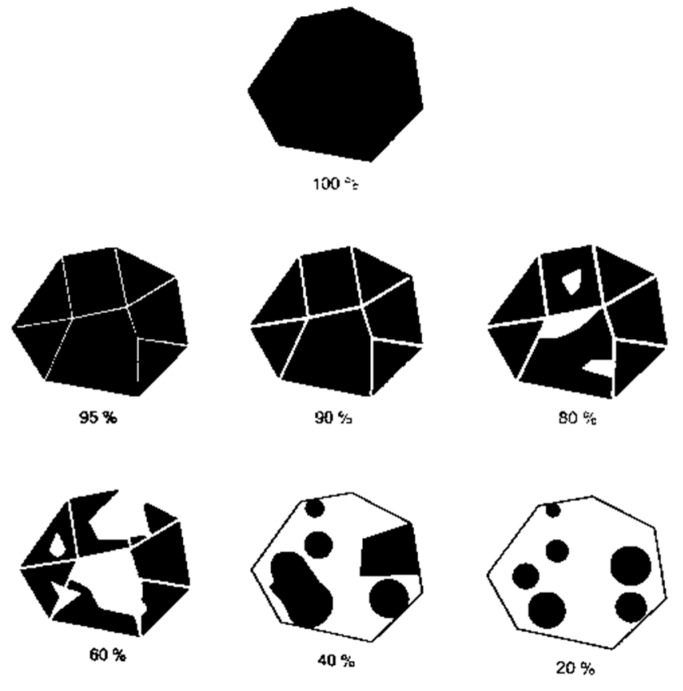
Visual references to evaluate degree of bitumen coverage.

After finishing the test, the particles were visually evaluated by two different persons separately; each one defined the coating percentage for each particle of aggregates based on the guidance presented in Figure 2, obtained from standard EN 12697-11 [35]. The results are expressed by an average of the individual evaluations.

In the first moment, all the mastics and bitumen were tested without the adhesion improver additives. However, only those mastics that did not achieve the minimum coverage area demanded by the standard (≥85%) after the adhesiveness test received an addition of 0.3 wt % of adhesion improver. The mastics that did not achieve the requirements after adding 0.3 wt % of adhesion improver were tested for 0.5 wt %. 

The mastic with 40% RM without additive, for example, achieved the coverage area requested, and it was not necessary to do the test with 0.3 wt % or 0.5 wt %

The percentage of adhesion improver is related to the total weight of bitumen used for producing the mastics. Table 4 shows the mastics that required chemical additives.

## 3. Results

### 3.1. Mastic Characterization

**Figure 3:**
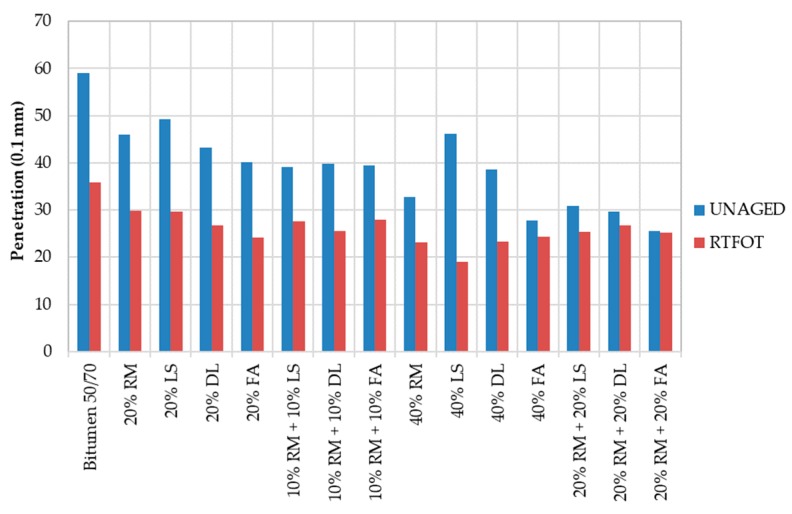
Penetration results for mastics before and after rolling thin film oven test (RTFOT).

Figure 3 shows the reduction in penetration after aging, which also indicates an increase in viscosity. In general, the gap between unaged and aged mastics decreases when the filler content increases. The more filler, the stiffer the mastic and the lower the effects of aging.

The 40% LS mastic has the biggest difference. The small size of limestone particles allows mastics to be better mixed and softer than those mastics with fly ash and red mud. On the other hand, after submitting the mastic into higher temperatures within the RTFOT test, the limestone promoted the polarization of bitumen molecules, increasing the viscosity of the sample.

**Figure 4:**
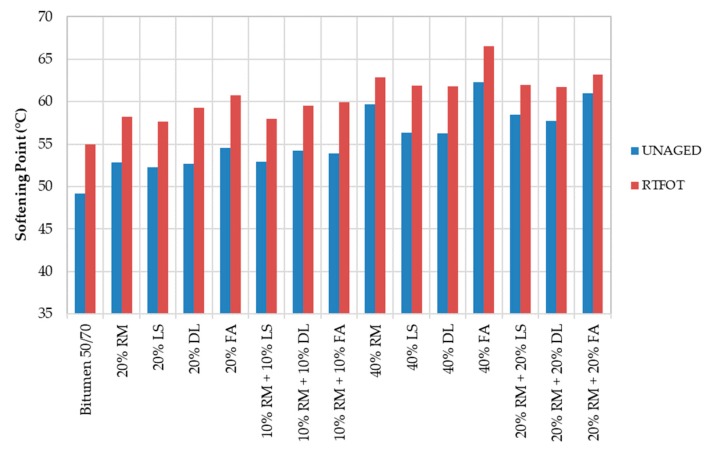
Softening point of mastics before and after RTFOT.

The softening points of the mastics are given in Figure 4. As shown, the more filler, the stiffer the material. The 40% LS also has a big gap between the unaged and aged results. However, in Figure 4, the 40% FA is shown to be the mastic with the greatest viscosity. In both Figure 3 and Figure 4, the reference bitumen presents the highest viscosity.

The thermal susceptibility indicates the sensibility of bitumen mastics to the temperature variation. Different approaches can be used to determine the thermal susceptibility of binders. Pfeiffer and Van Doormaal [36] proposed the thermal susceptibility index or penetration index for this purpose. This index is determined using the softening point and penetration at 25 °C, as shown in Equation (1):

(1) $$\mathrm{PI}=\frac{500\cdot \log\left(P\right)+20\cdot SP-1951}{120-50\cdot \log\left(P\right)+SP}$$

**Figure 5:**
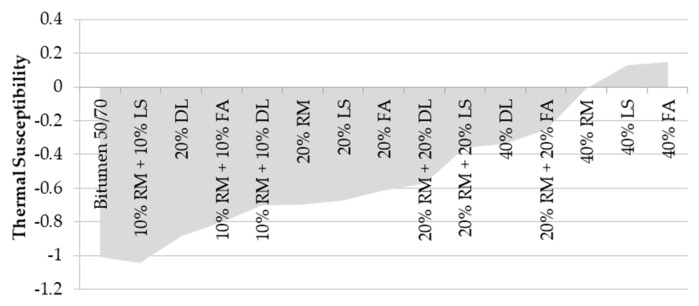
Thermal susceptibility index.

The lower the index, the lower the thermal susceptibility. The index ranges between −1.5 and +0.7. Values greater than +1 indicate oxidized asphalts (poorly sensitive to high temperatures); values lower than −2 indicate very temperature sensitive asphalts. Figure 5 shows the thermal susceptibility index calculated for the mastics.

The mastics with a higher content of filler have greater values than the others, which indicates lower sensibility to temperature variation due to the oxidation of the material. On the other hand, the reference bitumen mastics with a lower content of filler obtained negative values, indicating their sensibility to the temperature.

### 3.2. Wettability

**Figure 6:**
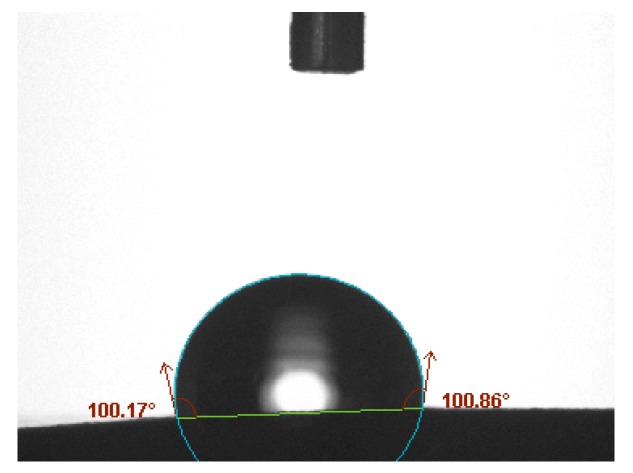
Contact angle in the 40% red mud (RM) mastic.

Figure 6 shows an example of measurement. 

The wettability results show (see Table 5) the dolomite as the more hydrophilic filler since the contact angle decreases when the amount of dolomite increases. The opposite occurs with the red mud. Thus, the contact angle increases as the mastic has more red mud, which characterizes the residue as a hydrophobic material.

The fly ash and limestone have less affinity to water than the dolomite, but more than the red mud. All mastics fabricated with two fillers achieved intermediate contact angles in comparison with the mastics composed with the same fillers isolated. This indicates that both fillers have an influence on the mastic’s properties.

### 3.3. Adhesive Test Results

**Figure 7:**
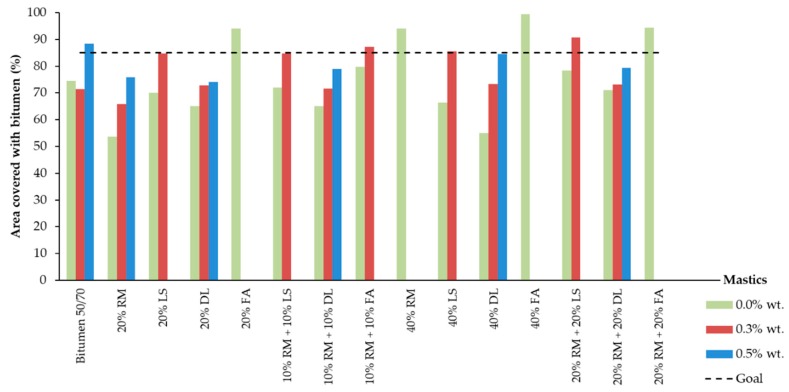
Covered area of aggregates after adhesiveness test (with and without additives).

Figure 7 shows the aggregates’ area covered with mastics/bitumen within different percentages of additive after the water sensibility test.

The only samples without additives that achieved the 85% required by the standard were the 20% FA, 40% RM, 40% FA, and 20% RM + 20% FA. The mastic with 10% RM + 10% FA almost reached the minimum value, with 80% of the aggregates’ area covered.

The results show the FA as the best filler to increase bonding properties between bitumen and granite aggregates. Even in a smaller amount, all the mastics with FA achieved good results in comparison with the other mastics.

In general, mastics with limestone and dolomite had results lower than the required level, as well as the reference bitumen 50/70.

The mastics that did not achieve the minimum requested by the standard were retested. This time, we added 0.3 wt % and 0.5 wt % of additive, considering the total amount of bitumen used to fabricate the mastics. Despite the small reduction observed in the reference sample, the more additive added, the greater the binding properties. 

**Figure 8:**
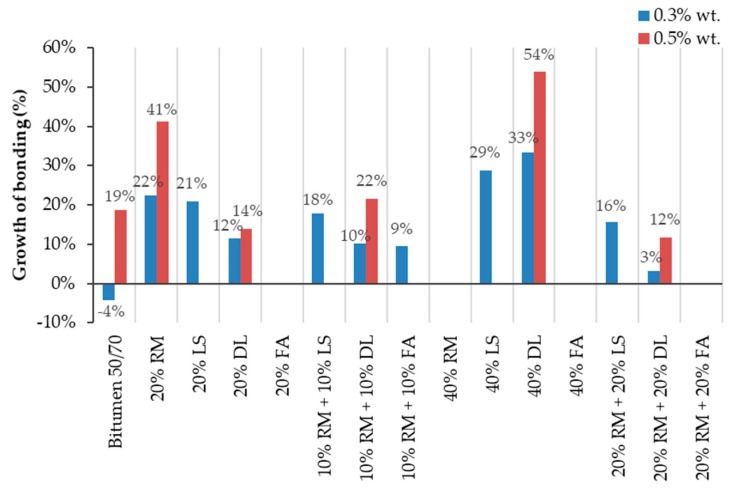
The growth rate of bonding properties relative to samples without an additive.

Figure 8 shows the growth of the bonding values after adding 0.3 and 0.5 wt % of additive, taking as reference the samples without additive. As shown, the highest influence of the additive occurred on mastics with dolomite and 20% RM.

Figure 7 and Figure 8 show that the more dolomite there is inserted in the samples without additive, the lower the adhesive properties. However, the addition of the additive promotes a wider improvement in the results the more dolomite the mastic has. This leads us toward two main conclusions—the dolomite by itself decreases the bonding properties between the film of bitumen and the granite aggregates, but when combined with additives, it can potentialize its effects.

The red mud and the dolomite isolated have shown great improvement in adhesiveness with 0.3% and 0.5% of additive. On the other hand, when with mixed the mastic, this mixture did not present a substantial improvement, remaining almost the same for the 20% and 40% samples (Figure 7). This could indicate that red mud and dolomite annul each other’s influences. In other words, the mastics with both dolomite and red mud are the average of the results of these fillers when isolated.

The addition of 0.3% additive within the mastics with limestone improved the results, but not in the same proportion as the amount of limestone inserted. Thus, it is not possible to state that the improvement in the adhesive properties is linked only to the proportion of limestone, being also due to the amount of additive inserted. The CaO presented in the limestone is responsible for increasing the bonding properties between the 20% LS and 40% LS mastics.

Within mastics with limestone, the red mud did not appear to significantly influence the adhesiveness properties, since the improvement in the results for those with 10% RM + 10% LS and 20% RM + 20% LS are alike. If mastics with 20% RM and 20% LS are compared with the 10% RM + 10% LS, it is possible to observe an improvement when compared with the 20% RM. On the other hand, if the 20% RM + 20% LS is compared with the 40% RM and 40% LS, it is possible to observe a larger influence from the red mud. In other words, for mastics with a smaller amount of red mud, the limestone seems to have more influence, but when the red mud has a higher content, its influence seems to be wider.

Within the mastics composed of red mud and fly ash in all contents, it is observed that those mastics with both at the same time have intermediate results between the performance of the fly ash and red mud isolated.

**Figure 9:**
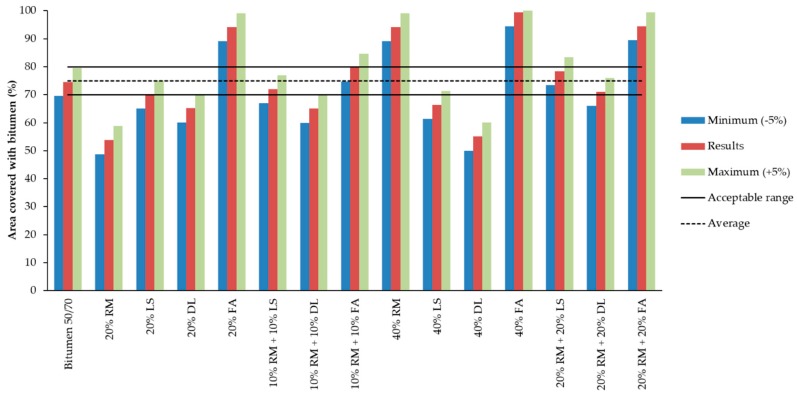
Covered area of aggregates considering a ±5% range (without additive).

As shown in Figure 8, the bitumen 50/70 had a 4% reduction in the coverage area after adding 0.3 wt % of adhesion improver. This reduction may be caused by the inaccuracy of visual analysis. In order to reduce the inaccuracy and observe which mastics had a greater performance within the adhesiveness properties, a 5% range of acceptance is used for the samples without additive, as shown in Figure 9. If ±5% is considered as an acceptable variation, it is possible to equalize the results obtained by the reference sample with 0 and 0.3 wt % additive. 

In Figure 9, it is possible to compare the results obtained with those for bitumen 50/70, which is considered the reference. Two additional columns were created considering the results obtained in the adhesiveness test. The superior column had +5% and the inferior column −5%. The reference lines are based on the bitumen 50/70 results. 

For comparison purposes, Table 6 shows a rating parameter designed to determine the mastics’ performance relative to the 50/70 bitumen without additive.

Considering the ±5% variation presented in Figure 9, only the 20% RM and 40% DL mastics would be considered inadequate, since their results are worse than the reference.

Most mastics had a greater or equal performance in comparison with the bitumen 50/70. Taking into account the fact that the results shown do not have additives, it is possible to attribute the mastics’ performance to the influence of the fillers. 

In Table 6, it is possible to observe a pattern among the mastics’ performance. All samples with fly ash are classified as ‘better’ than the reference. All samples composed with dolomite are ‘worse’ than the reference (except the one with 20% RM). The samples with limestone are in the middle and have a similar performance to the reference. 

The red mud is within all categories; however, in the samples combined with other fillers and within higher content (40% filler), the mastics presented an ‘equal’ or ‘better’ performance in relation to the reference. The mastics with red mud have a ‘better’ classification when mixed with fly ash, ‘worse’ when mixed with dolomite and are similar to the bitumen 50/70 when mixed with limestone.

**Figure 10:**
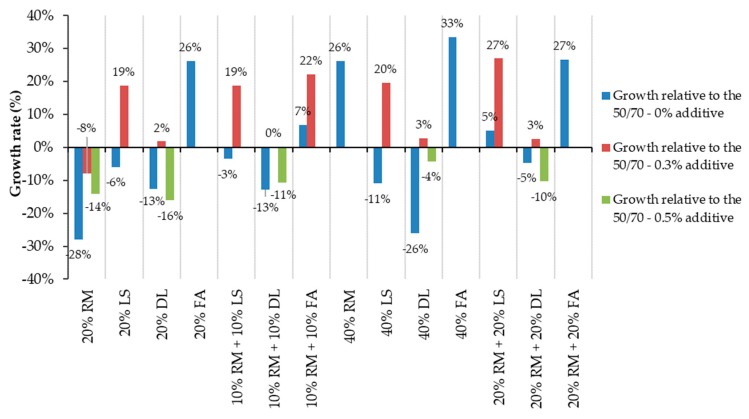
The growth rate relative to the reference sample.

Figure 10 shows the growth rate relative to the reference sample. Each mastic is compared with the appropriate bitumen 50/70 results (i.e., mastics without additive are also based on the reference without additive).

The growth rates are higher for those mastics without an additive and composed of fly ash (e.g., the 40% FA increased the adhesiveness by 33% considering the bitumen 50/70). The mastics with 20% FA, 40% RM, and 20% RM + 20% FA had the same rate of growth. The mastics with 20% RM and 40% DL had values 26–28% under the reference, which means that the growth was reduced. These reductions were lower when the additive is inserted. 

Although the reference sample has a small inaccuracy due to the negative growth (see Figure 7 and Figure 8), it is possible to observe that all the mastics with limestone substantially increased their adhesive properties by including 0.3% additive. This notable divergence of growth between mastics suggests limestone as an adhesive potentializing agent.

### 3.4. General Analysis of the Results

Bitumen comprises complex micelles of asphaltenes with a weak chemical affinity for aggregate, whereas the aggregate is characterized by a strong affinity for water [37]. Indeed, the granite aggregate used in this research naturally has a lower affinity for bitumen and requires anti-stripping additives to improve its bonding properties. To improve the affinity between materials, the adhesive agent promotes the polarity without changing any other properties of the bitumen (e.g., viscosity). 

As shown in the results, the additive helped to improve the adhesive properties of the 50/70 bitumen and mastics. However, calcium-based mastics had a greater improvement than others when combined with additives. Calcium-based fillers such as limestone and dolomite are commonly used to improve adhesive properties since they lower the interfacial energy between bitumen and mineral aggregates. In this study, the limestone and the dolomite obtained greater improvements after adding 0.3% and 0.5% of additives, which suggests calcium-based fillers as adhesive enhancers agents.

Despite the improvement promoted by the CaO, mastics with dolomite obtained the worst adhesive results. This happened due to the MgO in its composition that has an affinity to water and produces Mg(OH)_2_. This reaction promotes a volumetric expansion, which generates tensions that ruin the particle in pieces, allowing more water to come inside the mastic and remove the binder film from the aggregate. Thus, the adhesive properties of the dolomite are strongly dependent on its hydrophilic characteristics [38]. 

Unlike the carbonate fillers (dolomite and limestone), fly ash and red mud have a more diverse mineral composition. Thus, predicting their effects regarding properties such as surface characteristics and wettability is a more complex task.

The red mud, for example, is an alkaline-porous material without expansive clay minerals. The higher porosity of red mud positively influences the penetration of the bitumen and increases the adhesiveness. The presence of iron (Fe_2_O_3_) helps the red mud to adsorb surface-active substances from the crude oil, leading to a hydrophilic surface [39]. Its alkalinity and hydrophobicity also promote a strong bond with asphalt due to its acidic nature. Hence, mastics composed of red mud offer a satisfactory resistance against moisture permeation and had good active–passive adhesions [40].

The fly ash chemical composition attributes a more alkaline character that increases the indirect tensile strength (van der Waals forces), strengthening the physicochemical interactions between the bitumen surface and the fly ash particles. The percentage of SiO_2_ (>50%) did not worsen the water resistance. Woszuk et al. [40] reported that a higher content of unburnt carbon in the fly ash provides higher hydrophobicity, reducing the water sensibility. Indeed, the fly ash obtained good hydrophobic results that indicate a strong bonding with bitumen. According to Bautista et al. [41], the CaO content present in the fly ash indicates a greater volume of calcium sulfite hemihydrate (CaSO_3_), which may also contribute to the higher water resistance.

The bitumen has a complex system characterized by intermolecular associations of polar molecules (asphaltenes) dispersed in a more apolar continuous phase of saturated paraffin, aromatic oils, and resins called maltene [42]. The oxidation of some organic components occurs due to the losses of volatile substances and promotes the stiffness of the bitumen (aging).

This is normally attributed to polar molecular interactions [43,44]. The increasing number of asphaltenes causes the immobilization of an excessive number of polar molecules and, ultimately, bitumen embrittlement. In this case, the final asphalt is susceptible to fracturing after thermal or mechanical stress [42]. 

The additives used to reduce aging act on the inorganic–organic interfacial tension, dispersing the inorganic particles among the bitumen matrix. It is believed that the additive used in this study has an apolar nature that acts in the maltene phase, weakening asphaltene inter-cluster interactions and softening the bitumen [42]. In the first moment, the reduction of viscosity promotes a better covering of the aggregates by the mastics.

The raised stiffness of mastics was not necessarily caused by bitumen oxidation but by the addition of fillers. It is possible that a higher viscosity also plays a role in the adhesive properties. The connection between the aggregate and the bitumen is reinforced by the fillers, and higher viscosities reduce the possibility of water penetration, as they make the film around the aggregate less susceptible to temperature.

The mastics with the highest thermal susceptibility had the worst adhesiveness results (50/70 to 20% LS). This reinforces the correlation between mastic viscosity and bitumen–aggregate bonding potential. This statement is especially true for mastics composed of red mud and limestone. When it comes to fly ash and dolomite mastics, their performance is also influenced by the chemical composition, where MgO is responsible for worsening dolomite adhesiveness and CaO and SO_3_ improve the adhesiveness of fly ash mastics.

## 4. Conclusions

This study examines the adhesive properties of fillers and their influence when mixed with red mud. Thus, mastics composed of traditional fillers (limestone, dolomite) are compared with fly ash and red mud in different percentages.

Hence, the following points are concluded:

- The granitic rocks used in this work have electronegativity and hydrophilicity characteristics, which reduce the bonding properties between the bitumen and the aggregates in the presence of water, especially for mastics with dolomite.
- Calcium-based fillers potentialize the effects of the additive.
- The alkalinity and hydrophobicity attributed to the fly ash as well as the presence of CaO and SO_3_ in its composition are the main factors responsible for improving the affinity between the aggregates and bitumen.
- The additive used in this study has an apolar nature that acted in the maltene phase, weakening the asphaltene inter-cluster interactions and softening the bitumen.
- The viscosity of the mastics plays an important role in the adhesive properties.
- The lower alkalinity and the MgO within the dolomite composition significantly influence its affinity toward the bitumen.
- Larger amounts of red mud (≥40 vol % of bitumen) help to improve the hydrophobicity of mastics and, therefore, their adhesive properties.
- Red mud in smaller quantities (≤20 vol % of bitumen) has little influence over the adhesive properties of mastics.
- The combination of different fillers provides distinct mastic properties.
- The influence of fillers in the mastics is proportional to the amount inserted.

Despite widespread use, adhesive tests performed by visual analysis are often questioned by the scientific community because of their inaccuracy. Therefore, current methods should be improved to include more accurate analysis.

In fact, in future works, it is recommended to evaluate the adhesion of mastics with microscopic tests, as well as the properties of asphalt mixtures produced with alternative materials.

## Figures and Tables

**Table 1:** Filler characterization.

Characterization Tests	RM	DL	LS	FA	Standards
BET-Specific Surface Area (m^2^/g)	8.3	4.1	2.5	2.9	-
Density (g/cm^3^)	2.8	2.7	2.7	2.9	EN 1097-6 [22]
Rigden Voids (g/cm^3^)	44.8	30.9	32.6	42.5	EN 1097-4 [23]
Ph	12.4	6.76	7.8	13.1	ISO 10523 [24]
Size, d_10_ (μm)	1.1	2.4	2.5	4.1	-
Size, d_50_ (μm)	7.2	10.1	10.8	55.2	-
Size, d_90_ (μm)	120.1	38.1	39.6	63.0	-

**Table 2:** Chemical composition of fillers.

Chemical Composition	RM %	DL %	LS %	FA %
Fe_2_O_3_	29.86	0.34	-	1.15
Al_2_O_3_	21.96	0.64	0.54	5.03
SiO_2_	18.31	2.50	3.0	46.6
Na_2_O	11.33	0.82	-	1.22
TiO_2_	5.50	-	-	-
CaO	1.53	31.0	51.81	24.68
MgO	0.23	17.0	0.50	2.11
MnO	0.17	-	-	-
P_2_O_5_	0.10	-	-	-
SO_3_	0.09	-	0.11	8.26
K_2_O	0.07	0.76	-	4.35
Cr_2_O_3_	0.04	-	-	-

**Table 3:** Bitumen characterization.

Characterization Tests	50/70	Standard
Softening Point, °C	49	EN 1427 [26]
Penetration, 0.1 mm	59	EN 1426 [27]
Frass breaking point, °C	−15	EN 12593 [28]
Kinematic viscosity 135 °C, mm^2^/s	483.4	EN 12595 [29]
Dynamic viscosity 60 °C, Pa.s	60	EN 12596 [30]
Ductility	11.8%	EN 13398 [31]
RTFOT	-	EN 12607 [32]
Softening Point, °C	55	EN 1427 [26]
Penetration, 0.1 mm	36	EN 1426 [27]

**Table 4:** Samples that required adhesion improver agent.

Mastic/Bitumen	0.0 wt %	0.3 wt %	0.5 wt %
50/70	×	×	×
40% RM	×		
40% LS	×	×	
40% DL	×	×	×
40% FA	×		
20% RM + 20% LS	×	×	
20% RM + 20% DL	×	×	×
20% RM + 20% FA	×		
20% RM	×	×	×
20% LS	×	×	
20% DL	×	×	×
20% FA	×		
10% RM + 10% LS	×	×	
10% RM + 10% DL	×	×	×
10% RM + 10% FA	×	×	

**Table 5:** Samples that required adhesion improver agent.

Mastic/Bitumen	Contact Angle (°)
Bitumen 50/70	87.5
20% RM	83.5
20% LS	81.7
20% DL	78.3
20% FA	76.6
10% RM + 10% LS	80.8
10% RM + 10% DL	70.5
10% RM + 10% FA	79.0
40% RM	100.5
40% LS	76.3
40% DL	56.3
40% FA	72.1
20% RM + 20% LS	83.5
20% RM + 20% DL	94.6
20% RM + 20% FA	75.2

**Table 6:** Performance rating between mastic and reference sample (without additive).

*When?*	*Classification*	*Mastics*
All columns above the superior line	>(better)	20% FA
		40% RM
		40% FA
		20% RM + 20% FA
At least two columns (average and maximum) above the average line	≥(better or equal)	10% RM + 10% FA
		20% RM + 20% LS
At least one column (maximum) in the average line	=(equal)	20% LS
		10% RM + 10% LS
		20% RM + 20% DL
The ‘maximum’ column in the inferior line	≤(worse or equal)	20% DL
		40% LS
		10% RM + 10% DL
All columns under minimum line	<(worse)	20% RM
		40% DL
